# Predicting time to relapse in patients with schizophrenia according to patients’ relapse history: a historical cohort study using real-world data in Sweden

**DOI:** 10.1186/s12888-021-03634-z

**Published:** 2021-12-21

**Authors:** Kristian Tore Jørgensen, Martin Bøg, Madhu Kabra, Jacob Simonsen, Michael Adair, Linus Jönsson

**Affiliations:** 1 H. Lundbeck A/S, Ottiliavej 9, 2500 Valby, Denmark; 2 Otsuka Pharmaceutical Europe Ltd., Wexham, UK; 3 Department of Neurobiology, Care Sciences and Society, Karolinska Institutet, Solna, Sweden

**Keywords:** schizophrenia, relapse, methodology, real-world data

## Abstract

**Background:**

For patients with schizophrenia, relapse is a recurring feature of disease progression, often resulting in substantial negative impacts for the individual. Although a patient’s relapse history (specifically the number of prior relapses) has been identified as a strong risk factor for future relapse, this relationship has not yet been meticulously quantified. The objective of this study was to use real-world data from Sweden to quantify the relationship of time to relapse in schizophrenia with a patient’s history of prior relapses.

**Methods:**

Data from the Swedish National Patient Register and Swedish Prescribed Drug Register were used to study relapse in patients with schizophrenia with a first diagnosis recorded from 2006–2015, using proxy definitions of relapse. The primary proxy defined relapse as a psychiatric hospitalisation of ≥7 days’ duration. Hazard ratios (HRs) were calculated for risk of each subsequent relapse, and Aalen-Johansen estimators were used to estimate time to next relapse.

**Results:**

2,994 patients were included, and 5,820 relapse episodes were identified using the primary proxy. As the number of previous relapses increased, there was a general trend of decreasing estimated time between relapses. Within 1.52 years of follow-up, 50% of patients with no history of relapse were estimated to have suffered their first relapse episode. 50% of patients with one prior relapse were estimated to have a second relapse within 1.23 years (HR: 1.84 [1.71–1.99]) and time to next relapse further decreased to 0.89 years (HR: 2.77 [2.53–3.03]) and 0.22 years (HR: 18.65 [15.42–22.56]) for 50% of patients with two or ten prior relapses, respectively. Supplementary analyses using different inclusion/exclusion criteria for the study population and redefined proxies of relapse reflected the pattern observed with the primary analyses of a higher number of prior relapses linked with increased risk of/reduced estimated time to the next relapse.

**Conclusions:**

The results suggested a trend of accelerating disease progression in schizophrenia, each relapse episode predisposing an individual to the next within a shorter time period. These results emphasise the importance of providing early, effective, and tolerable treatments that better meet a patient’s individual needs.

**Supplementary Information:**

The online version contains supplementary material available at 10.1186/s12888-021-03634-z.

## Background

Schizophrenia is a chronic and relapsing mental disorder with wide-ranging impacts on affected people. Symptoms of schizophrenia can include hallucinations and delusions (‘positive’ symptoms) [1], social withdrawal and lack of emotional reactivity (‘negative’ symptoms), as well as cognitive impairments that can lead to functional decline. Despite relatively low global prevalence, schizophrenia was found to be the 15^th^ greatest cause of disability worldwide [2].

Relapse is a recurring feature of disease progression in schizophrenia, and is associated with substantial negative impacts for the individual, such as worsening symptoms, a deterioration in cognitive function and an overall decrease in quality of life [3, 4]. In addition, patients who suffer multiple relapse episodes may experience longer recovery times and have a decreased likelihood of regaining previous levels of health and functioning [5]. Relapse episodes can also induce a significant financial burden, including on the overall healthcare system [6, 7].

The most influential factor shown to increase a patient’s risk of relapse is non-­adherence to antipsychotic (AP) medication [3, 8]. Identifying and encouraging the use of tolerable pharmacological interventions, with the aim of increasing treatment adherence, is necessary to reduce the risk of relapse for patients with schizophrenia [5, 9–11]. Prior relapse has also been shown to be a strong predictor of future relapse [12].

Other factors that have been shown to increase risk of relapse include an individual’s history of depression, substance abuse and/or prior psychiatric hospitalisation [3, 13, 14]. In contrast, strong social skills and family support networks may reduce the risk of relapse for an individual [15, 16].

Studies using real-world data (RWD) are important to understand patterns of relapse in patients with schizophrenia under routine clinical care. Although relapse prevention in patients with schizophrenia is a main aim of clinical care and research, there are no standard criteria used to identify a schizophrenia relapse [5]. Relapse is frequently identified in patients following the worsening of symptoms, often leading to hospitalisation, although symptoms can vary between individuals [4, 5, 17]. The majority of retrospective studies do not have access to clinical information regarding a patient’s symptoms, and therefore database studies often rely on the use of proxies to identify relapse.

### Overview and objective

Despite evidence that prior relapse is a strong risk factor for future relapse, studies have not yet meticulously quantified the relationship between a patient’s relapse history (specifically the number of previous relapses) and the time to next relapse. The primary objective of this study was to use RWD from a cohort of patients diagnosed with schizophrenia in Sweden to quantify the relationship of time to relapse in schizophrenia with a patient’s history of prior relapses.

## Methods

### Swedish national registers

This was a historical cohort study based on data from two national registers of healthcare information in Sweden, maintained by the National Board of Health and Welfare (Socialstyrelsen). These are the Swedish National Patient Register and the Swedish Prescribed Drug Register, which contain data for the entire Swedish population.

The Swedish National Patient Register collects data on all hospitalisations since 1987 and psychiatric specialist outpatient visits since 2001 in Sweden. Demographic, geographic, administrative (both for inpatient and outpatient settings) and medical data are collected in this register [18]. From this register, we extracted data regarding schizophrenia diagnoses, hospitalisations and outpatient psychiatric specialist visits for the study population. A schizophrenia diagnosis during either a hospitalisation or outpatient psychiatric specialist visit was identified if there was a recorded International Classification of Diseases (ICD)-10 diagnosis code of F20.0–F20.9 [19], or an ICD-9 diagnosis code of 295.0–295.9, excluding 295.7 [schizoaffective disorder] [20]. A psychiatric hospitalisation was identified in the database if there was a diagnosis code from F01–F69 (ICD-10), F90–F98 (ICD-10) [19] or 290–316 (ICD-9) [20].

The Swedish Prescribed Drug Register collects data on all the prescribed drugs dispensed in pharmacies in Sweden and includes patient and prescriber demographic details as well as prescription information such as treatment duration and cost [21]. These data are available from 2005 onwards. In this register, drug information is categorised using the Anatomical Therapeutic Chemical (ATC) classification system. For this study, AP drugs were defined using the ATC code N05A [22]. For the individuals included in this study, we extracted data related to AP treatment over the study period.

Access to the Swedish databases used in this study was approved by the Regional Ethics Board of Lund (Dnr. 2018/775). The study is based on registry data. Informed consent of participation is therefore not applicable. All methods were carried out in accordance with relevant guidelines and regulations.

### Study population and follow-up period

For this research, relapse episodes were studied from 1 January 2006–31 December 2015. The register extracts included all patients with at least one schizophrenia diagnosis from 1987–2015 and with at least one filled AP drug prescription from 2005–2015. For inclusion in the study, patients had to meet the following criteria within the study period: at least two records of a schizophrenia diagnosis (which could include primary or secondary diagnoses, or diagnosis records from hospital attendance, either as an inpatient or outpatient), at least one filled AP drug prescription, and at least one discharge from a psychiatric hospitalisation. Patients were excluded from the primary analysis if they had received their first schizophrenia diagnosis or had their first discharge from a psychiatric hospitalisation prior to 2006, or were >60 years old at their first schizophrenia diagnosis. The start of follow-up for each patient was the date of discharge from their first psychiatric hospitalisation with ≥1 overnight stay during the study period. All patients who were discharged from their first psychiatric hospitalisation, regardless of schizophrenia diagnostic history, were initially included in the follow-up. These patients were then included in the subsequent analysis if they received two schizophrenia diagnoses over the course of the study period. The end of follow-up for each patient was the end of the study period or date of death, whichever occurred first.

Two supplementary analyses were conducted based on study populations with broader inclusion criteria. One analysis included all patients with ≥1 schizophrenia diagnosis and the other analysis included all patients who received their first schizophrenia diagnosis or first discharge from a psychiatric hospitalisation prior to 2006.

### Study procedures and evaluations

The primary outcome of interest in this study was the number and timing of relapse episodes. However, the clinical evidence of relapse, such as worsening of schizophrenia symptoms, is not directly recorded in the Swedish national registers. Therefore, a set of proxies were used to identify episodes of schizophrenia relapse. This study used a conservative primary proxy to identify relapse episodes, as well as two secondary proxies which identified additional relapse episodes.

### Primary proxy: psychiatric hospitalisation of at least 7 days’ duration

The primary proxy identified a relapse episode based on a psychiatric hospitalisation lasting ≥7 days. The relapse start date was the date of hospital admission and the relapse end date was the end of the first consecutive seven days after discharge, without rehospitalisation. If a patient was re-admitted to hospital within the first seven days after discharge, this would not be counted as another relapse episode but rather be grouped together as one episode.

In addition, a redefined primary proxy was used with stricter rehospitalisation criteria than the main primary proxy. This redefined proxy identified the relapse end date as the end of the first consecutive 30 days without rehospitalisation (instead of the first consecutive seven days).

### Supplementary analyses

#### Secondary proxies

Analyses were also conducted on two secondary proxy definitions of relapse, to understand if these different definitions of relapse identified a different relapse pattern compared to the primary proxy definition. The secondary proxy 1 included relapse episodes identified by a psychiatric hospitalisation with ≥1 overnight stay, followed by a switch in AP treatment. The switch in treatment was defined as the start of a new AP treatment line ≤30 days after hospital discharge. For this study, all first- and second-generation oral and long-acting injectable AP drugs were included as possible treatment lines. The secondary proxy 2 included relapse episodes that were defined based on a ‘high-frequency’ pattern of outpatient psychiatrist visits. ‘High-frequency’ was defined as a period of at least two consecutive weeks during which a patient had a minimum of eight outpatient psychiatry visits during the two-week period. The start date of the relapse episode was the end of this two-week period. The end date for the relapse episode was determined based on a reduction in the frequency of a patient’s outpatient visits to ≤1 per week for four consecutive weeks. For each of these redefined study populations, the other inclusion and exclusion criteria used for the primary analysis remained the same.

#### Inclusion of prevalent cases

As stated above, the main study population excluded patients who had received their first schizophrenia diagnosis or had their first discharge from a psychiatric hospitalisation prior to 2006. This supplementary analysis used the primary proxy for definition of relapse and included all patients in the databases with an existing schizophrenia diagnosis on 1 January 2006, as well as incident cases. For this analysis the study period was unchanged (1 January 2006–31 December 2015), and data regarding a patient’s prior psychiatric hospitalisations in the period from 1987–‍2005 were used to analyse the patient’s relapse history and time since first psychiatric hospitalisation.

### Statistical analysis

We report descriptive statistics for the study population including gender, age at first diagnosis and start of follow-up, follow-up duration, time pre-diagnosis, time post-diagnosis, and deaths during the study period. Since an individual can experience multiple relapse episodes, relapses were analysed as recurrent events.

Multivariate-adjusted Cox proportional hazards regression modelling was used to compare the risk of relapse in patients, with the number of prior relapses and age class (18–24, 25–29, 30–34, 35–39, 40–44, 45–49, 50–54, 55–59 and ≥60 years) as time-varying covariates. Additional Cox model covariates included gender and calendar year. The models estimated the cause-specific hazard of relapse and treated all other events (i.e. death or end of follow-up) as censoring events. Baseline risk was modeled as time since the end date of the first relapse (i.e. the end of the first consecutive seven days after discharge from first psychiatric hospitalisation without readmission). Hazard ratios (HRs) for the risk of each subsequent relapse were reported with 95% confidence intervals (CIs).

The Aalen-Johansen estimator [23] was used to estimate the risk of relapse, expressed as the time taken for a proportion of the population to experience their next relapse, whilst accounting for the competing risk of death. Calculations were completed for up to ten relapses. In this model, patients were grouped by number of prior relapses. The underlying time in the model was reset after the end date for each relapse episode (i.e. the end of the first consecutive seven days after hospital discharge without readmission), and the model did not take into account the length of the previous relapse episode. For the patients with no prior relapses at the start of follow-up, the time started from 0 on the date of discharge from their first psychiatric hospitalisation.

All analyses were performed using the statistical software SAS®, Version 9.4 or higher (SAS Institute Inc., Cary, NC, USA).

## Results

### Patient disposition and baseline characteristics

**Fig. 1:**
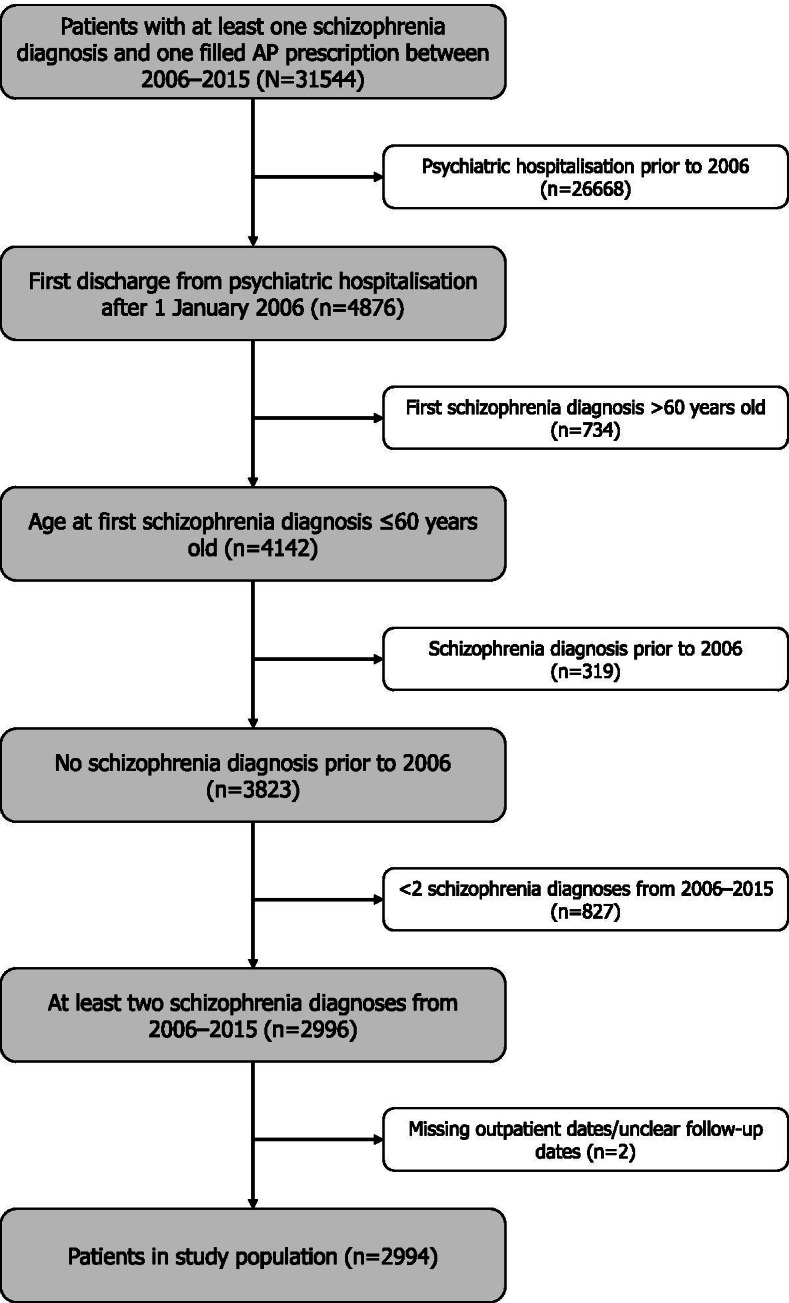
Study population flowchart AP: antipsychotic

A total of 31,544 patients had at least one schizophrenia diagnosis and one AP drug prescription between 1 January 2006 and 31 December 2015. Of these, 2,994 patients fulfilled the study inclusion criteria without meeting any exclusion criteria (Fig. 1). Patients had a mean (±standard deviation [SD]) age of 33.3 (±11.5) years at first diagnosis and a mean age of 32.4 (±12.0) years at the start of follow-up. The mean (±SD) duration of follow-up was 5.9 (±2.6) years. Around two thirds of the study population were male (66.4%) (Table 1). Over the course of the study period, there were 117 deaths (3.9%). A summary of the number of patients and relapse episodes identified using each proxy definition and for the supplementary analyses can be found in Supplementary Table S1.

**Table 1:** Baseline patient characteristics

Patient characteristics	Study population (n=2994)
**n (%)**	
Gender	
Male	1987 (66.4)
Female	1007 (33.6)
**Mean (±SD)**	
Age, years	
At first diagnosis	33.3 (±11.5)
At start of follow-up	32.4 (±12.0)
Follow-up duration, years	5.9 (±2.6)
Time pre-diagnosis, years	0.9 (±2.4)
Time post-diagnosis, years	5.1 (±2.7)

SD: standard deviation

### Primary proxy

**Fig. 2:**
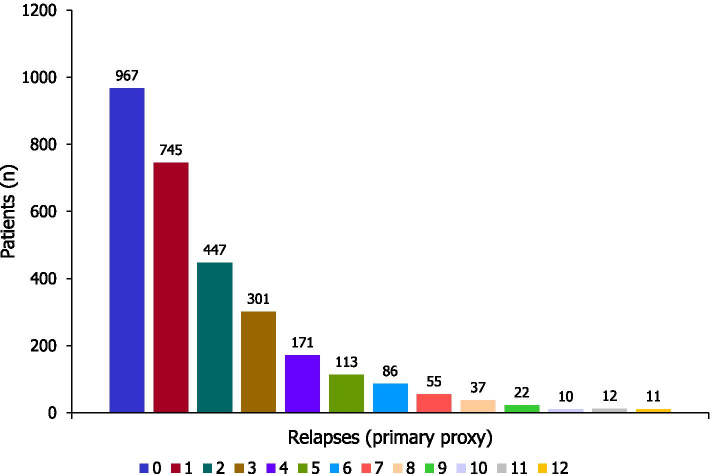
Number of patients by number of relapse episodes (primary proxy, 5820 episodes). Primary proxy: identified a relapse episode based on a psychiatric hospitalisation ≥7 days (n=2994)

**Fig. 3:**
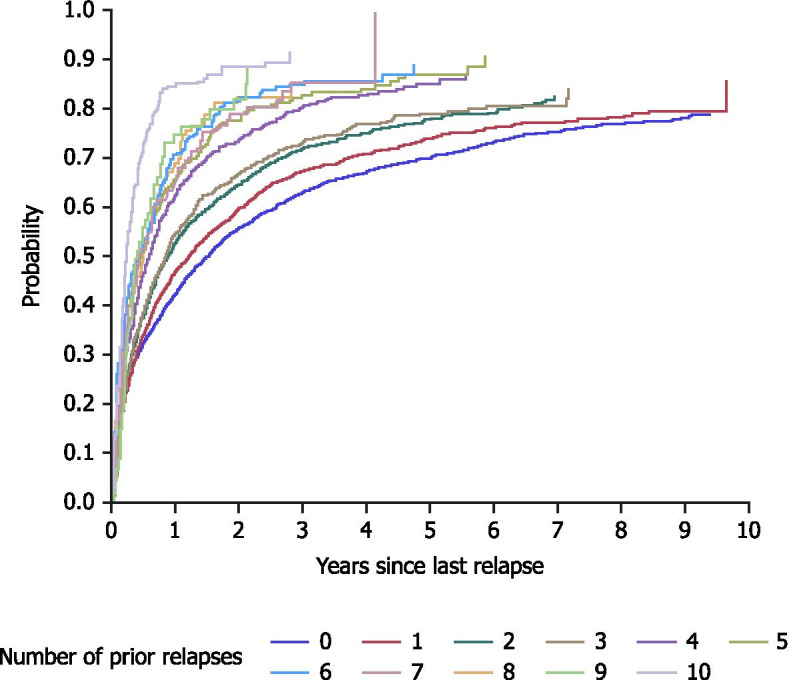
Estimated time to next relapse based on the number of prior relapses identified by the primary proxy. Aalen-Johansen plots were used to estimate the probability of relapse as a function of time since the last relapse. Primary proxy: identified a relapse episode based on a psychiatric hospitalisation ≥7 days (n=2994)

**Fig. 4:**
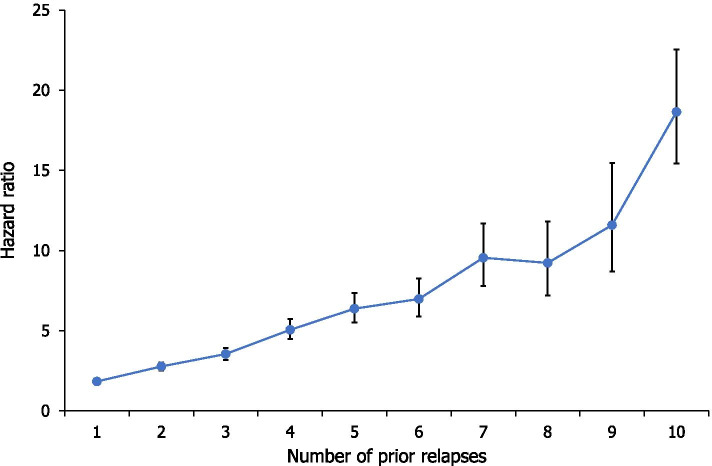
Hazard ratios based on number of prior relapses (primary proxy). Hazard ratios represent the hazard of relapse for those having the specified number of prior relapses relative to those with 0 relapses. Covariates in the Cox model included prior number of relapses, the calendar year, gender and age class at the start of follow-up. Primary proxy: identified a relapse episode based on a psychiatric hospitalisation ≥7 days (n=2994). Error bars represent 95% CIs. Statistical significance was estimated by the 95% CI. If the 95% CI included 1, then the time to relapse was not statistically significantly different from the reference group. If the 95% CI excluded 1, then time to relapse was significantly different from the reference group. CI: confidence interval

5,820 relapse episodes were identified using the primary proxy (Fig. 2). Of the 2,994 patients in the study population, 2,027 (67.7%) experienced at least one relapse episode as defined by this proxy, when identifying relapse events within the study period. Figure 3 shows an overall reduction in the estimated time to next relapse with increasing numbers of previous relapses. Within 1.52 years of follow-up, 50% of patients with no history of relapse (since the index hospitalisation) were estimated to have suffered their first relapse episode (Table 2). Subsequently, there was a general trend of decreasing estimated time between relapses. 50% of patients with one prior relapse episode were estimated to have a second relapse after 1.23 years, and this time to next relapse decreased further to 0.22 years for patients with ten prior episodes (Fig. 3; Table 2). Compared with patients with no history of relapse (following the index hospitalisation), the HR of relapse (95% CI) was 1.84 (1.71–‍1.99) for patients with one prior relapse episode and 2.77 (2.53–3.03) for patients with two prior relapses (Fig. 4; Table 2).

**Table 2:** Hazard ratios and time to next relapse by number of prior relapses (primary proxy and secondary proxy 1)

Number of prior relapses	Primary proxy			Secondary proxy 1		
	Patients, n^a^	Median time to next relapse, years	HR (95% CI)^b^	Patients, n^a^	Median time to next relapse, years	HR (95% CI)^b^
**0**	2994	1.52	-	2993	-	-
**1**	2024	1.23	1.84 (1.71–1.99)	779	6.47	2.76 (2.39–3.18)
**2**	1278	0.89	2.77 (2.53–3.03)	278	2.26	6.10 (5.00–7.43)
**3**	829	0.85	3.55 (3.19–3.94)	128	1.80	5.69 (4.32–7.48)
**4**	530	0.59	5.06 (4.48–5.73)	60	0.72	10.11 (7.03–14.54)
**5**	362	0.46	6.38 (5.53–7.37)	32	1.23	10.82 (6.77–17.31)
**6**	248	0.44	6.98 (5.89–8.27)	19	1.08	7.55 (3.72–15.32)
**7**	164	0.43	9.55 (7.79–11.70)	8	0.84	16.92 (6.95–41.22)
**8**	109	0.51	9.23 (7.21–11.81)	5	1.31	13.72 (4.37–43.11)
**9**	71	0.39	11.59 (8.69–15.47)	3	0.07	35.98 (11.34–114.20)
**10**	49	0.22	18.65 (15.42–22.56)	3	0.04	10.02 (2.48–40.48)

^a^After each relapse there was a censoring time of seven days after each event. Since not all patients could be followed after the time-window (due to the end of the follow-up period), the number of patients at risk of the n'th relapse was slightly smaller than the number of patients with the n'th-1 prior relapses

^b^Patients with no history of relapse (following the index hospitalisation) were used as the reference group in the Cox model. Primary proxy: identified a relapse episode based on a psychiatric hospitalisation ≥7 days. Secondary proxy 1: identified a relapse episode as a psychiatric hospital contact with >1 overnight stay, followed by a switch in AP treatment. Time to next relapse reflects the time by which 50% of patients would be estimated to have experienced their next relapse; this information was extracted from the Aalen-Johansen plot. Statistical significance was estimated by the 95% CI. If the 95% CI included 1, then the time to relapse was not statistically significantly different from the reference group. If the 95% CI excluded 1, then time to relapse was significantly different from the reference group. AP: antipsychotic; CI: confidence interval; HR: hazard ratio

When the redefined primary proxy was analysed (relapse end date as the end of the first consecutive 30 days without rehospitalisation), a total of 5,125 relapse episodes were identified (compared with 5,820 for the original primary proxy). 1,984 patients (66.4%) experienced at least one relapse episode as defined by this proxy (n=2,990), when identifying relapse events within the study period. Compared with patients who had no prior relapse, the HR of relapse (95% CI) was 1.75 (1.62–1.89) for patients with one prior relapse and 2.58 (2.35–2.84) for patients with two prior relapses.

### Supplementary analyses

#### Secondary proxies

The secondary proxy 1 identified 1,318 relapse episodes. 780 patients (26.1%) experienced at least one relapse episode as defined by this proxy, when identifying relapse events within the study period (n=2,993; one patient was excluded from this relapse count due to having a treatment switch just before the start of follow-up. They subsequently did not complete the required censoring time of seven days after each event before the start of follow-up). Supplementary Figure S1 shows that the estimated time to next relapse decreased for patients with a higher number of previous relapses. Based on this model, 50% of patients with one prior relapse episode were likely to have a second relapse within 6.47 years, and this decreased to 2.26 years for a third relapse (Supplementary Figure S1; Table 2). Compared with patients with no prior relapse, the HR of relapse (95% CI) was 2.76 (2.39–3.18) for patients with one prior relapse and 6.10 (5.00–7.43) for patients with two prior relapses (Supplementary Figure S2; Table 2). The secondary proxy 2 only identified four relapse episodes and, therefore, was not included in the analysis.

#### Inclusion of prevalent cases

A total of 28,863 patients were eligible for the analysis when we included patients with a schizophrenia diagnosis and/or first discharge from a psychiatric hospitalisation before 1 January 2006. Patients had a mean (±SD) age of 36.1 (±11.5) years at first diagnosis (N=28,860). The mean (±SD) duration of follow-up was 18.7 (±8.4) years (N=28,863). Over half of this study population was male (58.6%). Using the primary proxy, 150,627 relapse episodes were identified for this study population. Compared with patients with no prior relapse, the HR of relapse (95% CI) was 2.02 (1.98–2.06) for patients with one prior relapse and 3.05 (2.98–3.12) for patients with two prior relapses.

#### Inclusion of patients with a single schizophrenia diagnosis

A total of 3,821 patients were eligible for the analysis when patients with ≥1 schizophrenia diagnosis were included. Patients had a mean (±SD) age of 33.4 (±11.6) years at first diagnosis. The mean (±SD) follow-up duration was 5.8 (±2.7) years. Around two thirds of the study population were male (65.4%). Over the course of the study period, there were 158 deaths (4.1%) in this study population. The primary proxy identified 7,116 relapse episodes in this population. Compared with patients who had no prior relapse, the HR of relapse (95% CI) was 1.94 (1.81–2.07) for patients with one prior relapse and 2.90 (2.67–‍3.14) for patients with two prior relapses.

## Discussion

Relapse history has been shown to be a strong predictor of subsequent relapses in patients with schizophrenia [12], although the temporal relationship between recurrent relapses has not been well documented. This retrospective cohort study, based on data extracted from Swedish national registers, suggests that there is a correlation between the time to a patient’s next schizophrenia relapse and the number of previous relapses they have had. The results showed that as the number of prior relapses increased, the risk of a subsequent relapse (quantified using HRs) also increased, alongside decreasing estimated time to relapse. The results point to a pattern of accelerating disease progression in schizophrenia, where each additional relapse episode predisposes an individual to the next.

Furthermore, this pattern of increasing risk and decreasing time to a subsequent relapse continued to be observed for patients with increasing numbers of prior relapses. For patients with five prior relapses, 50% of individuals were estimated to have their next relapse within 0.46 years (approximately five and a half months), and the estimated time to next relapse decreased to 0.22 years (approximately two and a half months) for patients with ten prior relapses. Similarly, the HR (95% CI) of relapse increased from 6.38 (5.53–7.37) to 18.65 (15.42–22.56) for patients with five or ten prior relapses, respectively.

In addition to the primary proxy definition for relapse, the secondary proxy 1 was used to identify relapse episodes in the study population, defining relapse as ≥1 night of psychiatric hospitalisation and an AP treatment switch. This analysis was conducted to compare how different proxy definitions of relapse (≥7 days inpatient hospitalisation versus ≥1 inpatient hospitalisation followed by an AP treatment switch) may affect the observed pattern of relapse in the same study population. The analyses using the primary proxy and secondary proxy 1 produced different values for estimated time between relapses. However, a similar pattern of relapse was observed using the two proxy definitions: a higher number of prior relapses was linked to an increased risk of subsequent relapse and a reduced estimated time to the next relapse (Supplementary Figure S3; Table 2). The comparability of these results, despite the different criteria used to define relapse for each proxy, is important for the validity of the overall findings and future implications of this study.

The results from this study provide supporting evidence that early in the schizophrenia disease progression there is, on average, a longer time between episodes of relapse. Therefore, early access to treatment that is both efficacious and tolerable may have a proportionally greater benefit in promoting sustained remission as compared with treatment provided later in the disease progression. The results from this study support previous research that showed preventing relapse early in a patient’s schizophrenia disease progression is crucial to sustaining positive long-term outcomes [8, 24, 25].

### Strengths and limitations

#### Study population

In this study, around two thirds of the study population were male (66.4%) (Table 1) and this is similar to that of other studies, where it has also been found that a higher proportion of patients in incident schizophrenia populations are male [26]. In contrast, some studies looking at *prevalent* schizophrenia populations have found no difference in the proportions of males and females [26–28].

Schizophrenia is typically first diagnosed in adolescence and early adulthood, with a peak prevalence and disease burden observed between 30 and 40 years old [27]. Despite these common observations, the incident patient population used for the primary analyses in this study had a mean age of 32.4 years at the start of follow-up, and a mean age of 33.3 years at first diagnosis (Table 1). The observation that the study population had a lower mean age of follow-up than mean age at first diagnosis is likely reflective of the inclusion criteria by which all patients who were discharged from their first psychiatric hospitalisation, regardless of schizophrenia diagnostic history, were initially included in the follow-up. These patients were then included in the final analysis if they received two schizophrenia diagnoses over the course of the study period.

The relatively high mean age of patients with schizophrenia in Sweden has been noted before in other studies that have extracted data from the Swedish national registers [29]. For the patients in this study, age at first psychiatric hospitalisation (the event used here to signify the start of follow-up) and the age at first diagnosis may occur later in life than a patient’s first schizophrenia symptoms. Diagnostic data from primary care were not available and, therefore, the management of patients with early symptoms in primary care could not be captured. In addition, a higher mean age of diagnosis (as compared to the mean age at the start of follow-up) may reflect a cautionary approach to diagnosing an individual with schizophrenia [29]. Lack of migration information in the national registers may also mean that older individuals who have received a schizophrenia diagnosis before migrating to Sweden will be classified as more recently diagnosed patients in the register [29]. Finally, it is important to note that the study population can be classified as a survivor cohort, in that only patients who survived to have at least one AP prescription recorded in the Swedish Prescribed Drug Register since 2005 were included.

#### Swedish national registers

The national patient registers in Sweden maintain an almost complete longitudinal history of patients with schizophrenia managed within the Swedish healthcare system. Therefore, these databases provide valuable insight into long-term patterns of relapse. A limitation of these national registers is that they do not contain information regarding clinical information that could indicate relapse such as worsening of symptoms or patient-reported/clinical quality of life assessments (such as scores on the Global Assessment of Functioning [GAF] scale). However, it is often the case that such assessments are conducted infrequently, and therefore would not fit within the granular timescale required for this research. As alluded to above, a further limitation of the Swedish national registers is that they do not include data on patient migration, which may lead to individuals being included in this incident study population despite having received a schizophrenia diagnosis before migrating to Sweden.

#### Follow-up period

The follow-up period for each patient started from the date of discharge from their first psychiatric hospitalisation with ≥1 overnight stay during the study period, whether their hospitalisation was due to schizophrenia or another psychiatric condition. Since all patients in the cohort were eventually diagnosed with schizophrenia, this index date was chosen to identify when the patient most likely had their first severe clinical symptoms of schizophrenia and to ensure that the calculation of time to first relapse and time to subsequent relapses were comparable both within and between individuals. A limitation of this approach is that patients who were only or primarily treated in an outpatient setting may not be included, or included late in the study population. An alternative index date would have been the time of a patient’s first schizophrenia diagnosis, in an inpatient or outpatient setting. However, this approach was not chosen as it would reduce the comparability between time to first relapse and time to second relapse. In addition, it would have resulted in patients enrolled at an older age compared with the current index date definition, as there can be a long delay between a person’s first symptoms and receiving a schizophrenia diagnosis. It is also important to note that the minimum length of stay for this index episode (≥1 overnight stay) differs from the definition of relapse used by the primary proxy (≥7 days inpatient hospitalisation) because this index episode was used to signify the onset of schizophrenia for each patient, rather than to identify a relapse. As previously mentioned, a supplementary analysis was conducted that redefined relapse as ≥1 night of psychiatric hospitalisation and an AP treatment switch (secondary proxy 1). The results of this analysis were similar to that of the primary proxy, showing a pattern of increasing risk of subsequent relapse and reducing estimated time to the next relapse.

#### Patient inclusion criteria

Prevalent cases were not included in the main analysis to avoid bias resulting from misclassification of the time of the first psychiatric hospitalisation. As a result, this study population is an ‘early in disease’ population (mean follow-up duration 5.9 years, with a maximum ten years’ follow-up by the end of the study period), as compared with the overall population of patients with schizophrenia in Sweden. A supplementary analysis that included all patients who had received their first schizophrenia diagnosis or had their first discharge from a psychiatric hospital prior to or after 2006 was conducted in order to increase the generalisability of the results to the wider schizophrenia patient population. The inclusion of prevalent patients in the analysis produced a similar pattern of results to the primary analysis. Therefore, it is expected that the relationship between time to next relapse and number of prior relapses is not only representative for patients relatively early in their schizophrenia disease, but is a general phenomenon in schizophrenia as a whole.

Similarly, an analysis of the population that included all patients with ≥1 schizophrenia diagnosis was performed to determine if this study population had a different risk pattern to the population specified for the primary analysis (requiring at least two schizophrenia diagnoses). The HRs were similar for groups with patients with ≥1 or ≥2 schizophrenia diagnoses.

#### Proxy definitions

A systematic literature review revealed that hospitalisation was the most common feature of proxy definitions used to identify schizophrenia relapse [3]. However, these studies often did not specify hospitalisation duration or type, or used terms that were not clearly defined or consistent between studies (e.g. ‘generic’ or ‘psychiatric’ hospitalisation and/or ‘partial’ or ‘full’ hospital stays) [3]. Our study used a primary proxy which defined a schizophrenia relapse episode specifically as a period of psychiatric hospitalisation ≥7 days. It is not possible to determine if during these hospitalisations patients were displaying clinical evidence of a relapse (i.e. worsening symptoms), or if the hospitalisation was the result of another, unrelated cause. However, using ICD codes to identify a psychiatric hospitalisation, as well as setting the minimum duration at ≥7 days, increased the likelihood that the included hospitalisations were the result of a true schizophrenia relapse. A limitation to the primary proxy definition for relapse is that it would not have captured relapse episodes with a shorter hospitalisation (<7 days), or episodes managed entirely in an outpatient setting.

The analyses in this study did not take into account the length of the previous relapse episode, which for some patients may continue for several months. The results from the primary proxy showed that an estimated 50% of patients had their first relapse within 1.52 years. After an additional 1.23 years, 50% of patients with one relapse had a second relapse (Table 2). With a mean follow-up time in this study of 5.9 (±2.6) years, there was sufficient follow-up time to capture multiple relapse episodes for a large number of patients, irrespective of the length of the individual relapse episodes.

As the primary proxy defines a relapse episode as a period of hospitalisation lasting ≥7 days, it is possible that the results indicating that the period between relapse episodes tends to decrease over time is, in part, reflective of ‘revolving door’ patients, i.e. those patients with a high frequency of short-term hospitalisations [30]. In a supplementary analysis, the primary proxy was redefined with stricter hospitalisation criteria, extending the censor period between hospitalisations to 30 days after each relapse instead of seven days. Therefore, the time period within which a rehospitalisation was attributed to the same relapse episode increased. The analyses that used this redefined primary proxy demonstrated the same relationship as the primary analysis, one of increasing risk associated with each additional prior relapse a patient has experienced. Therefore, it is unlikely that the results observed in this study are exclusively influenced by ‘revolving door’ patients.

#### Use of the Aalen-Johansen estimator

A strength of this study is that Aalen-Johansen plots were used to avoid the bias inherent in Kaplan-Meier estimators, in which death is a competing event for relapse (i.e. if death occurs then relapse can no longer occur).

## Conclusions

Understanding the likely trajectory of future relapses could help facilitate care planning and support treatment decisions to reduce the disease burden on patients with schizophrenia. This study has shown that there is a clear relationship between a patient’s relapse history and subsequent relapses, in which patients have a higher risk and a shorter estimated time to next relapse with higher numbers of prior relapses. This was confirmed in several supplementary analyses testing various definitions of relapse and patient inclusion criteria, as well as when we adjusted for important confounding factors such as age at diagnosis and sex. This study provides further evidence of the progressive nature of schizophrenia, and emphasises the importance of providing early, effective, and tolerable treatment options that better meet a patient’s individual needs, to enable treatment adherence, recovery and prevent future relapses, which is consistent with previous research [5, 9, 10, 14, 25, 31].

## Supplementary Information

**Additional file 1.**

**Additional file  2.**

**Additional file 3.**

**Additional file 4.**

**Additional file 5.**

## Data Availability

The data that support the findings of this study are available in the Swedish National Patient Register [https://www.socialstyrelsen.se/en/statistics-and-data/registers/register-information/the-national-patient-register/] and the Swedish Prescribed Drug Register [https://www.socialstyrelsen.se/en/statistics-and-data/registers/register-information/the-swedish-prescribed-drug-register/].

## Abbreviations

AP: Antipsychotic
ATC: Anatomical Therapeutic Chemical
CI: Confidence interval
GAF: Global Assessment of Functioning
HR: Hazard ratio
ICD: International Classification of Diseases
RWD: Real-world data
SD: Standard deviation
