# Supplementary material for: Predicting time to relapse in patients with schizophrenia according to patients’ relapse history: a historical cohort study using real-world data in Sweden
Source: BMC Psychiatry. 2021 Dec 21;21:634. doi: 10.1186/s12888-021-03634-z (PMC8690369; doi:10.1186/s12888-021-03634-z)
Supplement: Supplementary file 1 — Additional file 1. [file 12888_2021_3634_MOESM1_ESM.docx]

**SUPPLEMENTARY MATERIALS**

**Supplementary Table S1. Patients and relapse episodes identified by each proxy definition and supplementary analysis**

^a^Only 4 events were identified using the secondary proxy 2 alone and therefore this proxy definition was not included in the analysis; ^b^Using primary proxy definition of relapse. Primary proxy: identified a relapse episode based on a psychiatric hospitalisation ≥7 days. Secondary proxy 1: identified a relapse episode as a psychiatric hospital contact with ≥1 overnight stay, followed by a switch in AP treatment. Secondary proxy 2: defined a relapse episode as a period of at least two consecutive weeks during which a patient had a minimum of eight outpatient psychiatry visits. Redefined primary proxy: identified the relapse end date as the end of the first consecutive 30 days without rehospitalisation (instead of the first consecutive seven days). Inclusion of a single schizophrenia diagnosis: included all patients with ≥1 schizophrenia diagnosis (primary analysis required ≥2 schizophrenia diagnoses). AP: antipsychotic; N/A: not applicable; SD: standard deviation.

Supplementary Figure S1. Estimated time to next relapse based on number of prior relapses (secondary proxy 1)

Aalen-Johansen plots were used to estimate the probability of relapse as a function of time since the last relapse. Secondary proxy 1: identified a relapse episode as a psychiatric hospital contact with ≥1 overnight stay, followed by a switch in AP treatment (n=2993). AP: antipsychotic.

Supplementary Figure S2. Hazard ratios based on number of prior relapses (secondary proxy 1)

Hazard ratios represent the hazard of relapse for those having the specified number of prior relapses relative to those with 0 relapses. Covariates in the Cox model included prior number of relapses, the calendar year, gender and age class at the start of follow-up. Secondary proxy 1: identified a relapse episode as a psychiatric hospital contact with ≥1 overnight stay, followed by a switch in AP treatment (n=2993). Error bars represent 95% CIs. Statistical significance was estimated by the 95% CI. If the 95% CI included 1, then the time to relapse was not statistically significantly different from the reference group. If the 95% CI excluded 1, then time to relapse was significantly different from the reference group. AP: antipsychotic; CI: confidence interval.

Supplementary Figure S3. Hazard ratios based on number of prior relapses (primary proxy and secondary proxy 1)

Hazard ratios represent the hazard of relapse for those having the specified number of prior relapses relative to those with no prior relapses. Covariates in the Cox model included prior number of relapses, the calendar year, gender and age class at the start of follow-up of the patient. Primary proxy: identified a relapse episode based on a psychiatric hospitalisation ≥7 days (n=2994). Secondary proxy 1: identified a relapse episode as a psychiatric hospital contact with ≥1 night stay, followed by a switch in AP treatment (n=2993). AP: antipsychotic.

SUPPLEMENTARY TEXT

*Frailty model*

An analysis was conducted to test whether hazard ratios increased linearly, using a gamma frailty model.

A linear gamma frailty model rejected the hypothesis that individual heterogeneity could explain the pattern observed (p=0.0105) and therefore indicated that there is an effect of prior relapses on time to future relapse.

The use of the frailty model was important to identify whether the HR patterns for relapse observed were a result of individual heterogeneity in risk, or, that suffering a relapse makes an individual more susceptible to subsequent relapse. The results of the frailty model showed that the risk of future relapse was not completely the result of individual heterogeneity. Although conclusions regarding causation based on observational research should be made with caution, the results indicate that prior relapses are at least partly driving the increasing relapse risk over time.

**Secondary proxy 1: treatment line definition**

A treatment line is defined as a period in which a patient is continuously treated with a stable drug regimen for ≥6 weeks. In order to identify a genuine treatment change, a patient is regarded to have started a treatment line when they have filled prescriptions for ≥42 days’ drug supply without any intermediate treatment gaps >30 days. As such, the treatment line start date is the first prescription fill date. A switch in treatment is characterised by a patient initiating treatment using a different AP drug to the one used in the immediately preceding treatment line. During follow‑up, it is not uncommon for patients to have multiple changes in AP treatment lines.

**Cox proportional hazards modelling: additional details**

In the Cox models, the underlying baseline risk for a subsequent relapse episode was assumed to operate on time since disease occurrence. Since the time of first disease occurrence cannot be observed directly in this study, the model uses the patient’s first psychiatric hospitalisation (≥1 overnight stay). To calculate the hazard ratio for relapse by age, patients were grouped according to their age at the start of follow-up. When calculating the hazard ratio for a second relapse, patients were grouped based on age at the end of their first relapse, and so on. In the Cox model, the baseline risk was modelled non-parametrically, whereas covariates (the number of previous relapses, age, age at diagnosis, gender and calendar year), are assumed to enter log-linearly. Calendar year and age were treated as class variables by year (or as a class variable in 5-year intervals) to capture changes in psychiatric practice and care.
