# Supplementary material for: Predicting time to relapse in patients with schizophrenia according to patients’ relapse history: a historical cohort study using real-world data in Sweden
Source: BMC Psychiatry. 2021 Dec 21;21:634. doi: 10.1186/s12888-021-03634-z (PMC8690369; doi:10.1186/s12888-021-03634-z)
Supplement: Supplementary file 2 — Additional file 2. [file 12888_2021_3634_MOESM2_ESM.docx]

**Supplementary Table S1.** Patients and relapse episodes identified by each proxy definition and supplementary analysis

| **Proxy/supplementary analysis** | **Patients, n** | **Relapse episodes** | | | |
| --- | --- | --- | --- | --- | --- |
|  |  | **Total, n** | **Mean (±SD)** | **Minimum** | **Maximum** |
| Primary proxy | 2994 | 5820 | 1.9 (±2.5) | 0 | 30 |
| Secondary proxy 1 | 2993 | 1318 | 0.4 (±1.0) | 0 | 12 |
| Secondary proxy 2^a^ | N/A | 4 | N/A | N/A | N/A |
| Redefined primary proxy | 2990 | 5125 | 1.7 (±2.1) | 0 | 19 |
| Inclusion of patients with a single schizophrenia diagnosis^b^ | 3821 | 7116 | 1.9 (±2.5) | 0 | 30 |

^a^Only 4 events were identified using the secondary proxy 2 alone and therefore this proxy definition was not included in the analysis; ^b^Using primary proxy definition of relapse. Primary proxy: identified a relapse episode based on a psychiatric hospitalisation ≥7 days. Secondary proxy 1: identified a relapse episode as a psychiatric hospital contact with ≥1 overnight stay, followed by a switch in AP treatment. Secondary proxy 2: defined a relapse episode as a period of at least two consecutive weeks during which a patient had a minimum of eight outpatient psychiatry visits. Redefined primary proxy: identified the relapse end date as the end of the first consecutive 30 days without rehospitalisation (instead of the first consecutive seven days). Inclusion of a single schizophrenia diagnosis: included all patients with ≥1 schizophrenia diagnosis (primary analysis required ≥2 schizophrenia diagnoses). AP: antipsychotic; N/A: not applicable; SD: standard deviation.
