# Supplementary figures and images for: Predicting time to relapse in patients with schizophrenia according to patients’ relapse history: a historical cohort study using real-world data in Sweden
Source: BMC Psychiatry. 2021 Dec 21;21:634. doi: 10.1186/s12888-021-03634-z (PMC8690369; doi:10.1186/s12888-021-03634-z)

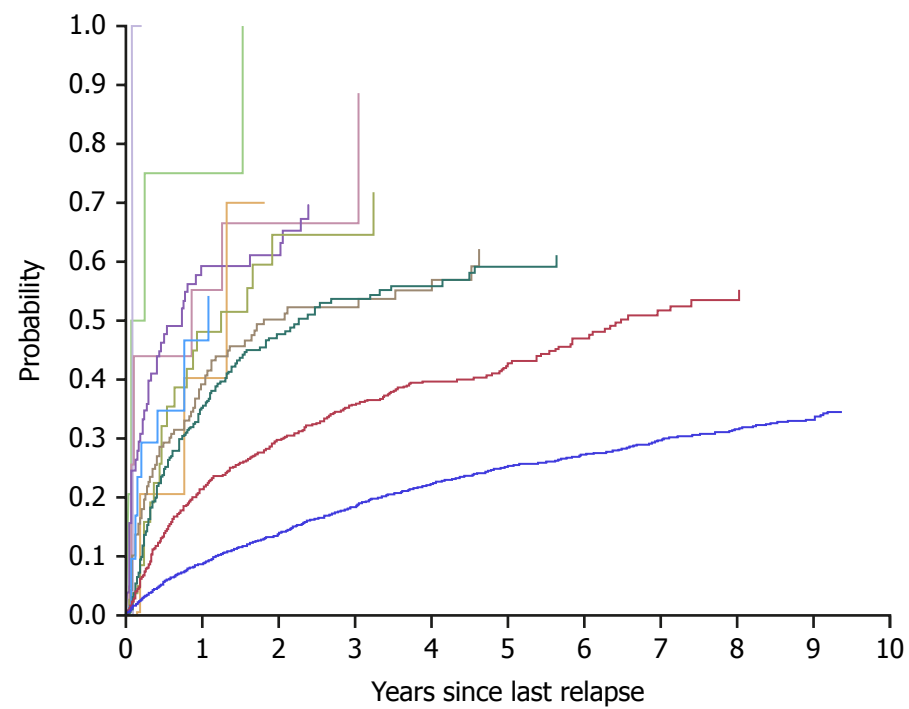

Number of prior relapses

|   |   |   |   |    |   |
|---|---|---|---|----|---|
| 0 | 1 | 2 | 3 | 4  | 5 |
| 6 | 7 | 8 | 9 | 10 |   |

Supplement: Supplementary file 3 — Additional file 3. [file 12888_2021_3634_MOESM3_ESM.pdf]

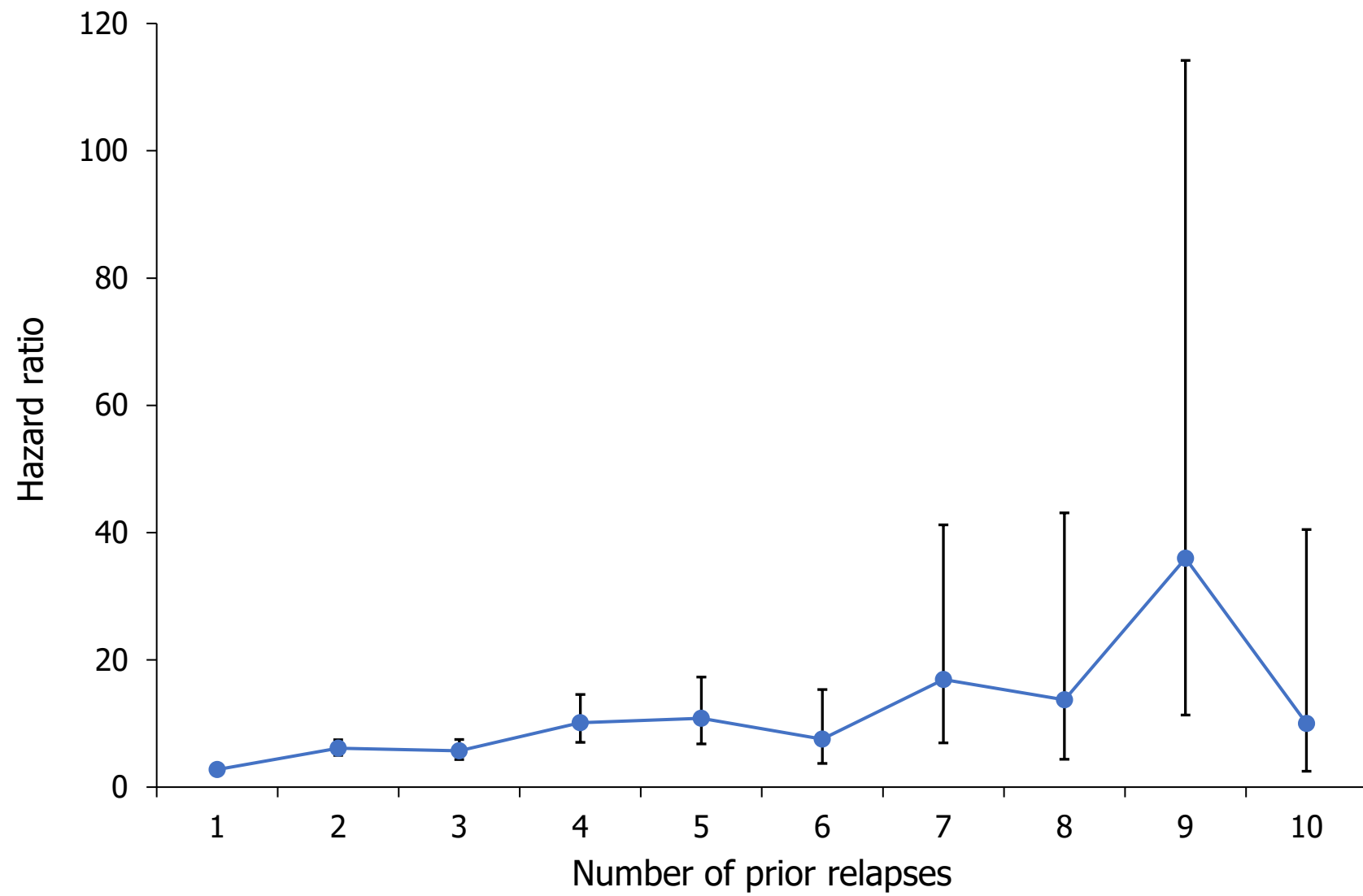

Supplement: Supplementary file 4 — Additional file 4. [file 12888_2021_3634_MOESM4_ESM.pdf]

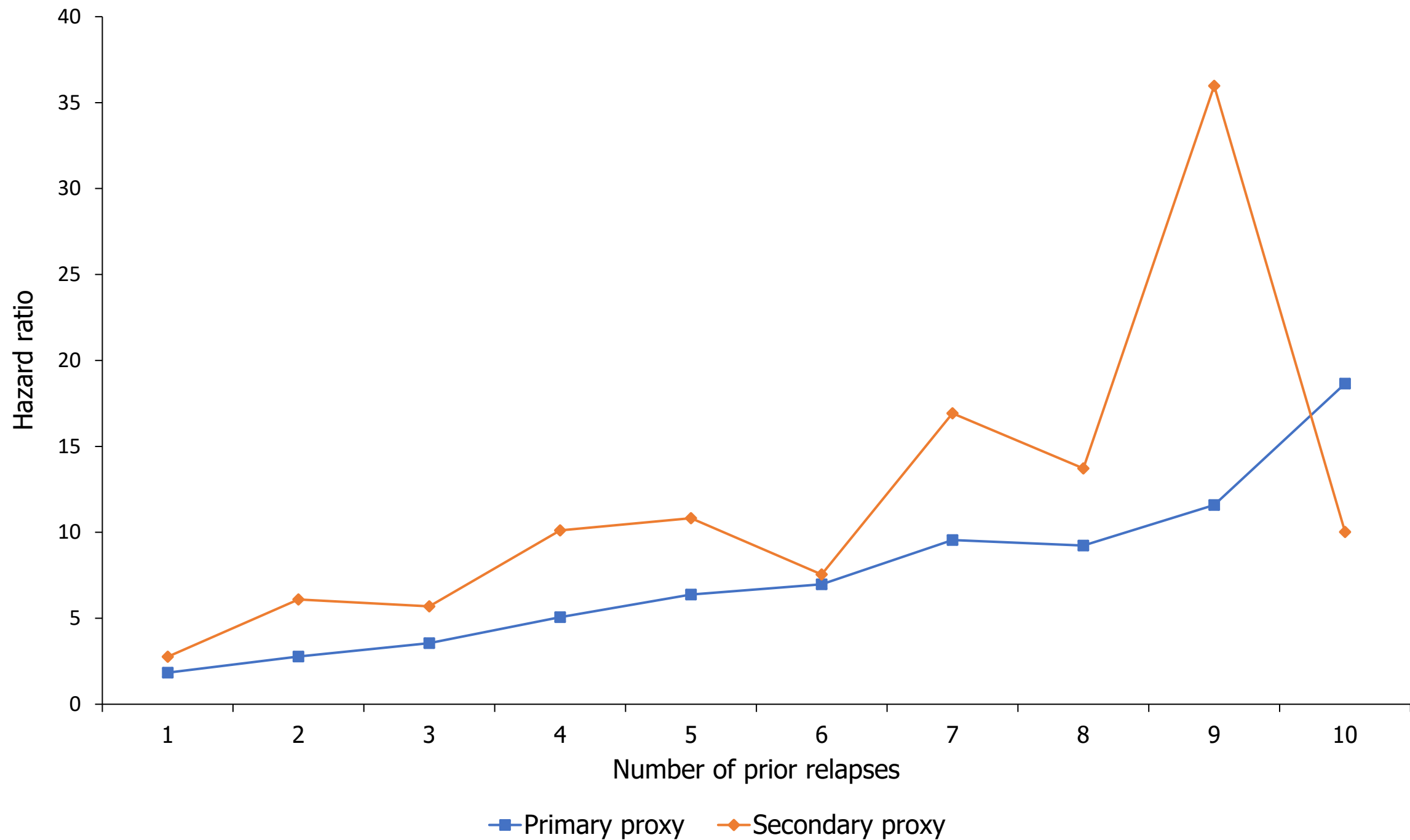

Supplement: Supplementary file 5 — Additional file 5. [file 12888_2021_3634_MOESM5_ESM.pdf]
